# 

**Published:** 1859-07

**Authors:** 


NORTH AMERICAN

### MEDICO-CHIRURGICAL REVIEW.

Vol. III.] JULY, 1859. [No. 4.

### CONTENTS.

### ANALYTICAL AND CRITICAL REVIEWS.

ART. PAGE

I.—1. A Treatise on Human Physiology. By J. C. DALTON, Jr., M.D. 577
2. Outlines of Physiology. By JOHN HUGHES BENNETT, M.D. . 577
3. Cours de Physiologie Comparée. Par M. FLOURENS . . 577
4. Leçons sur la Physiologie et l'Anatomie Comparée, etc. Par H. MILNE EDWARDS, M.D. . . . . . . . . 577
5. Cours de Médecine du Collége de France. Leçons sur la Physiologie et la Pathologie du Système Nerveux. Par M. CLAUDE BERNARD . . . . . . . . 577

II.—The Microscope, in its Application to Practical Medicine. By LIONEL BEALE, M.D., etc. . . . . . . . 611

III.—1. The Virginia Springs, and Springs of the South and West. By J. J. MOORMAN, M.D. . . . . . . . . . . 615
2. A Treatise on Baths, etc. By JOHN BELL, M.D. . . . 615

IV.—A Treatise on Diseases of the Air-Passages: comprising an Inquiry and Treatment of Bronchitis, Chronic Laryngitis, Clergyman's Sore-Throat, etc. By HORACE GREEN, M.D. . 628

V.—Upon a Function of the Pancreas but little known; the Digestion of Nitrogenous Food. By LUCIEN CORVISART, M.D. . . . 636

VI.—Five Essays. By J. K. MITCHELL, M.D. . . . . . . 640

### ORIGINAL COMMUNICATIONS.

I.—Contributions to Obstetric Jurisprudence. (No. 4.) By HORATIO R. STORER, M.D. . . . . . . . . . . . 643

II.—The Present State of Microscopical Science, Medically Considered. By JOHN H. PACKARD, M.D. . . . . . . . 658

III.—Proceedings of the Pathological Society of Philadelphia . . 677

IV.—The True Cause of Long and Short-Sightedness; with Observations on the Power to Dilate and Contract the Pupil of the Eye. By CHARLES W. WRIGHT, M.D. . . . . . 704

V.—Case of Cæsarean Section, in which both the Mother and Child were saved. By W. F. McCLELLAND, M.D. . . . . 707

### BI-MONTHLY ABSTRACTS.

#### SURGERY.

I.—Congenital Extrophy of the Urinary Bladder . . . . 709
II.—Cases of Popliteal Aneurism . . . . . . . 711
III.—Treatment of Hydrocele by Electricity . . . . . 712
IV.—Treatment of Chronic Hydrocephalus by Injections of Iodine . 713
V.—On Extirpation of Fibroid Tumors of the Uterus . . . 714
VI.—On the Treatment of Prolapsus Ani in Children . . . . 715

#### PATHOLOGY AND THERAPEUTICS.

I.—Anatomical and Physiological Researches on Dropsy Consecutive to Typhoid Fever . . . . . . . . . 716
II.—On the Existence of Albuminuria in Yellow Fever . . . . 718

ART. PAGE

III.—On the Use of Tannin in Large Doses in Albuminous Anasarca . 718
IV.—On Uræmia . . . . . . . . . . . . . 719
V.—A Case of Splenic Leucocythæmia . . . . . . . 721
VI.—On Nervous Vertigo . . . . . . . . . . . . 722
VII.—Contributions to the History of Nervous Diseases of Syphilitic Origin . . . . . . . . . . . . . 725
VIII.—On the Treatment of Chronic, Organic Diseases of the Heart . 726
IX.—Observations on the Diagnosis of Diseases of the Pancreas . 727
X.—On Oxalate of Lime in the Sediments of the Urine . . . . 728
XI.—Asbestus as an Irritant Application to the Skin . . . . . 729
XII.—Easy and Certain Cure of Facial Neuralgia . . . . . . 730
XIII.—Gunpowder, a Remedy for Toothache and for Scabies . . . 730
XIV.—On the Therapeutic Action of Solanin and of Dulcamara . . 731
XV.—Employment of Iodoform as an Anæsthetic Agent . . . . 731
XVI.—On the use of Alkalies as a Means of Obtaining the Extractive Principles of Vegetables . . . . . . . . 732
XVII.—Antidote to Phosphorus . . . . . . . . . . . 733

DISEASES OF WOMEN AND CHILDREN.

I.—New Theory and Treatment of Chlorosis . . . . . . 733
II.—On the Hypertrophic Elongation of the Cervix Uteri . . . 734
III.—On the Origin of Flexions of the Uterus . . . . . . 736
IV.—On Inflammation of the Fallopian Tubes as a Cause of Puerperal Peritonitis . . . . . . . . . . . . . 737
V.—Observations on Diphtheritic Angina and Laryngitis in Children . 739

PHYSIOLOGY.

I.—Rhythmical Contractions of a Voluntary Muscle of the Right Side 740
II.—Cause of the Tendency of Red-blood Corpuscles to arrange themselves in Piles . . . . . . . . . . . . 741
III.—Relative Temperature of the Submaxillary Saliva and of the Blood of the same side . . . . . . . . . . . 741
IV.—On the Influence of Exercise over Respiration and Pulsation . 742
V.—Glucogenic Material a Condition of Development of Tissues in the Fœtus, etc. . . . . . . . . . . . . 742
VI.—Physiology of Secretion . . . . . . . . . . 743
VII.—Effects of Ligature of Œsophagus in Animals . . . . . 744
VIII.—Amyloid Corpuscles as Normal Productions on the Skin . . 746

BI-MONTHLY PERISCOPE.

SURGERY.

1. Popliteal Aneurism Treated by Compression . . . . . . 747
2. On Labial Cancer . . . . . . . . . . . . . 749

PRACTICE OF MEDICINE.

Management of the Shoulders in Examinations of the Chest . . . 751

OBSTETRICS.

On Compression of the Aorta in Uterine Hemorrhage . . . . . 753

EDITORS' TABLE.—American Medical Association . . . . . . 755
Quarantine and Sanitary Convention . . . . . . . . 761
Medical Convention for Revising the Pharmacopœia of the United States.. . . . . . . . . . . . . . 763
Medical Society of the State of Pennsylvania.—Hospital Appointments . . . . . . . . . . . . . . . 764
Changes in Medical Journals.—Navy Medical Appointments.—Army Medical Appointments.—Chicago Medical Colleges . . . 765
Re-interment of the Remains of Dr. John Hunter . . . . . 766

NECROLOGICAL NOTICE.—Dr. William M. Boling . . . . . 766

BI-MONTHLY BIBLIOGRAPHICAL RECORD . . . . . . . . 768

